# Multiple sequence types responsible for healthcare-associated *Acinetobacter baumannii* dissemination in a single centre in Egypt

**DOI:** 10.1186/s12879-019-4433-1

**Published:** 2019-10-07

**Authors:** Leena Al-Hassan, Mai M. Zafer, Hadir El-Mahallawy

**Affiliations:** 1 Department of Global Health and Infection, Brighton and Sussex Medical School, G.19 Medical Research Building Brighton, Brighton, BN1 9PX UK; 2 Department of Microbiology and Immunology, Faculty of Pharmacy, Ahram Canadian University, Giza, Egypt; 3 Department of Clinical Pathology, National Cancer Institute, Cairo University, Cairo, Egypt

**Keywords:** *Acinetobacter baumannii*, Epidemiology, MLST, Carbapenem resistance

## Abstract

**Background:**

*Acinetobacter baumannii* is an increasingly worrying organism in the healthcare setting, due to its multidrug resistance and persistence. Prolonged hospitalisation, immunocompromised patients and excessive antibiotic exposure all contribute to increasing the risk of *A. baumannii* infections, which makes cancer patients a significant risk group. This study aims to investigate the dissemination of *A. baumannii* at the National Cancer Institute (NCI) in Cairo – Egypt.

**Methods:**

All bacterial isolates were typed using Multi-locus Sequence Typing (MLST) to characterise the epidemiology of isolates. The intrinsic OXA-51-like, and the acquired carbapanemases OXA-23, − 24/40, − 58, NDM, IMP, and VIM were also amplified and sequenced to genetically identify mechanisms of carbapenem resistance.

**Results:**

MLST results show a high degree of multi-clonal dissemination, with 18 different Sequence Types (STs) identified, including 5 novel. The majority of isolates belonged to International Clone (IC) 2, and carbapenem resistance was detected in 93% of isolates and mediated by *bla*_OXA-23_, *bla*_OXA-58_, *bla*_NDM-1_ and *bla*_VIM-1_. We also report the presence of a resistant ST732 (OXA-378) which has been previously identified in migratory birds_._

**Conclusions:**

Multiple highly resistant clones were identified in a Cancer hospital in Cairo. It is vital that clinicians and healthcare workers are aware of the population of *A. baumannii* present in order to have appropriate treatment and infection control practices.

## Background

*Acinetobacter baumannii* is a problematic organism commonly found as a persistent, multi-drug resistant organism in the hospital environment and causing a wide range of infections in patients with prolonged hospitalisation and severe comorbidities [1]. *A. baumannii* is also one of the ESKAPE pathogens (*Enterococcus faecium*, *Staphylococcus aureus*, *Klebsiella pneumoniae, Acinotobacter baumannii, Pseudomonas aeruginosa,* and *Enterobacter sp.*), which effectively “escape” the effects of current antibacterial drugs, thereby limiting therapeutic options [2]. Cancer patients are at a significant risk of acquiring *A. baumannii* due to many risk factors such as prolonged hospitalisation, being immunocompromised after chemotherapy treatment, and prophylactic administration of antibiotics [3, 4]. In Egypt, several studies have been published on the epidemiology of *A. baumannii* and other infections in hospitalised patients, but few studies have been published on cancer patients specifically. Furthermore, previous studies from Egypt have had limited molecular typing techniques conducted, which is very important to get an accurate epidemiological picture and spread of the organism [4–9]. We have previously investigated the molecular epidemiology of *A. baumannii* in 2013 in paediatric cancer patients in Egypt [9]. The aim of this study was to use Multi-locus Sequence Typing (MLST) to characterise the epidemiology of *A. baumannii* isolates from the National Cancer Institute (NCI) in Cairo - Egypt, to determine the sequence types present, and also genetically characterise carbapenem resistance.

## Methods

Fifty-nine non-repetitive *A. baumannii* isolates were collected in 2015 & 2016 from the NCI in Cairo, Egypt. Initial identification was performed at the NCI using VITEK 2 (bioMérieux, Marcy l’Etoile, France). Antibiotic susceptibility was determined using VITEK 2 and results were interpreted according to CLSI guidelines [10]. The gyrB multiplex method, previously described by Higgins et al. was used to confirm the species of isolates due to the very similar phenotypic characteristics of the *A. baumannii-calcoaleticus* complex [11]. All isolates were typed using the MLST Oxford scheme: briefly, 7 house-keeping genes (*gltA, gyrB, rpoD, cpn60, gdhB, gpi, recA)* were amplified, sequenced and assigned an allele number. The allele numbers are combined to yield a specific sequence type (ST) using the pubMLST database https://pubmlst.org/abaumannii/. The intrinsic *bla*_OXA-51-like_ gene, and the acquired carbapanemases OXA-23, − 24/40, − 58, NDM, IMP, and VIM for all isolates were also amplified and sequenced as mentioned previously [9, 12, 13].

## Results

Fifty-five isolates were confirmed *A. baumannii* in addition to 4 *A. pittii* (also known as Acinetobacter Genomic Species (GS) 3). Ten different variants of the intrinsic *bla*_OXA-51-like_ genes were identified: OXA-66, OXA-69, OXA-91, OXA-94, OXA-64, OXA-51, OXA-132, OXA-100, OXA-378, and a newly identified OXA-510 which shares 99% identity to OXA-64, (genbank accession number KU710721.1). The 4 *A. pittii* isolates did not harbour *bla*_OXA-51-like_ genes.

Interestingly, 5 isolates harboured *bla*_OXA-378_ as their intrinsic *bla*_OXA-51-like_ gene, which has been reported in stork nestlings in Poland [14], and recently in a clinical sample of a respiratory infection in Egypt (GenBank accession number LC382028.1). No further information is available on the isolate identified in Egypt, but the stork nestling isolates were carbapenem sensitive, whereas the current clinical isolates at the NCI were carbapenem resistant and contained *bla*_OXA-23_. All the NCI isolates were clonally related and assigned as ST732.

MLST typing revealed 18 different sequence types (for 30 *A. baumannii* isolates), 5 of which were newly assigned (ST 1577–1581) in the current study. *A. pittii* isolates were also typed with MLST and a single ST was assigned to all four isolates: ST1089. STs were unobtainable for 29 isolates due to the inability to amplify/sequence the *gdhB* and/or *gpi* loci recombination and insertion elements (IS) frequently disrupt these loci in the Oxford MLST scheme [15]. The largest group was part of International Clones (IC) 2 clone, Clonal Complex (CC) 92 containing 20 isolates. Table 1 lists all the 18 STs identified in the study. Five new STs were identified (ST1577–1581) and when comparing them to other STs in the database, ST1578 and ST1579 are Single-locus variants (SLV) of several STs belonging to IC2/CC92, whereas ST1581 is a Double-locus variant (DLV) to STs in IC4/CC103. No SLV or DLV were found for ST1577 containing OXA-132 as the intrinsic OXA-51-like enzyme.

**Table 1:** Summary of all isolates, carbapenem sensitivity, intrinsic OXA-51-like, Class D and B Carbapenemases, and Sequence Type* novel ST identified

Isolate number	Species	Carbapenem susceptibility	OXA-51-like gene	Class D-Carbapenemase (OXA-23, − 40, − 58)	MBL	ST
ABNCI - 6	*A. baumannii*	R	OXA-66	OXA-23		ST195
ABNCI - 10	*A. baumannii*	R	OXA-69	OXA-23		Unobtainable
ABNCI - 11	*A. baumannii*	S	OXA-94			ST1115
ABNCI - 12	*A. baumannii*	R	OXA-94	OXA-23	VIM-1	Unobtainable
ABNCI - 13	*A. baumannii*	R	OXA-94	OXA-23	VIM-1	ST957
ABNCI - 14	*A. pittii*	R	-ve	OXA-23		ST1089
ABNCI - 15	*A. baumannii*	R	OXA-66	OXA-23		Unobtainable
ABNCI - 2	*A. baumannii*	R	OXA-378	OXA-23		ST732
ABNCI - 25	*A. baumannii*	R	OXA-66	OXA-23		Unobtainable
ABNCI - 26	*A. baumannii*	R	OXA-66	OXA-23		ST195
ABNCI - 3	*A. baumannii*	R	OXA-66	OXA-23		Unobtainable
ABNCI - 29	*A. baumannii*	S	OXA-378	OXA-23		ST732
ABNCI - 30	*A. pittii*	R	-ve	OXA-23		ST1089
ABNCI - 32	*A. pittii*	R	-ve	OXA-23		ST1089
ABNCI - 33	*A. baumannii*	R	OXA-66	OXA-23		Unobtainable
ABNCI - 35	*A. baumannii*	R	OXA-66	OXA-23		Unobtainable
ABNCI - 36	*A. baumannii*	R	OXA-51-like	OXA-23		ST1581*
ABNCI - 37	*A. baumannii*	R	OXA-94	OXA-23		ST1580*
ABNCI - 38	*A. baumannii*	R	OXA-64	OXA-23		Unobtainable
ABNCI - 39	*A. baumannii*	S	OXA-51-like			ST1115
ABNCI - 4	*A. baumannii*	R	OXA-66	OXA-23		Unobtainable
ABNCI - 40	*A. baumannii*	R	OXA-66	OXA-23		Unobtainable
ABNCI - 41	*A. baumannii*	R	OXA-94	OXA-23	VIM-1	Unobtainable
ABNCI - 42	*A. baumannii*	R	OXA-51-like	OXA-23		Unobtainable
ABNCI - 43	*A. baumannii*	R	OXA-51-like	OXA-23		ST1100
ABNCI - 47	*A. baumannii*	R	OXA-94	OXA-23		Unobtainable
ABNCI - 48	*A. baumannii*	R	OXA-100	OXA-23		ST1100
ABNCI - 49	*A. pittii*	R	-ve	OXA-23		ST1089
ABNCI - 5	*A. baumannii*	R	OXA-51-like	OXA-23		Unobtainable
ABNCI - 50	*A. baumannii*	R	OXA-66	OXA-23		Unobtainable
ABNCI - 8	*A. baumannii*	R	OXA-66	OXA-23		Unobtainable
ABNCI - 9	*A. baumannii*	R	OXA-66	OXA-23		Unobtainable
NCI-1	*A. baumannii*	R	OXA-66	OXA-23	VIM-1, NDM-1	ST1579*
NCI-2	*A. baumannii*	R	OXA-94	OXA-23		ST1580*
NCI-3	*A. baumannii*	S	OXA-510			Unobtainable
NCI-4	*A. baumannii*	R	OXA-378	OXA-23		ST732
NCI-5	*A. baumannii*	R	OXA-66	OXA-23	VIM-1, NDM-1	ST286
NCI-6	*A. baumannii*	R	OXA-66	OXA-23	NDM-1	ST1578*
NCI-7	*A. baumannii*	R	OXA-378	OXA-23		ST732
NCI-8	*A. baumannii*	R	OXA-66	OXA-23	NDM-1	Unobtainable
NCI-9	*A. baumannii*	R	OXA-378	OXA-23		ST732
NCI-10	*A. baumannii*	R	OXA-91	OXA-23		Unobtainable
NCI-11	*A. baumannii*	R	OXA-69	OXA-23	NDM-1	ST441
NCI-13	*A. baumannii*	R	OXA-51-like	OXA-23		ST195
NCI-14	*A. baumannii*	R	OXA-64	OXA-23		Unobtainable
NCI-15	*A. baumannii*	R	OXA-66	OXA-23		ST208
NCI-16	*A. baumannii*	R	OXA-66	OXA-23		ST208
NCI-18	*A. baumannii*	R	OXA-51-like	OXA-23		ST236
NCI-19	*A. baumannii*	S	OXA-100			ST930
NCI-20	*A. baumannii*	R	OXA-91	OXA-23		Unobtainable
NCI-23	*A. baumannii*	S	OXA-132			ST1577*
NCI-24	*A. baumannii*	S	OXA-51			Unobtainable
NCI-25	*A. baumannii*	R	OXA-66	OXA-23		ST195
NCI-26	*A. baumannii*	R	OXA-94	OXA-23	NDM-1	ST1078
NCI-27	*A. baumannii*	R	OXA-100	OXA-58	NDM-1	ST472
NCI-28	*A. baumannii*	R	OXA-51-like	OXA-23	VIM-1	ST236
NCI-29	*A. baumannii*	R	OXA-66	OXA-58	NDM-1	Unobtainable
NCI-32	*A. baumannii*	R	OXA-69	OXA-23		NA
NCI-33	*A. baumannii*	R	OXA-94		NDM-1	ST1078

Carbapanem resistance was detected in 55 isolates (93%), and only 4 isolated were sensitive to carbapenem (Table 1). The most common class-D carbapenemase present in the isolates was OXA-23 present in 92.7% of *A. baumannii* (*n* = 51) and 100% of *A. pittii* isolates. Six isolates co-harboured VIM-1_,_ and 5 isolates co-harboured NDM-1. Two isolates contained OXA-23, NDM-1, and VIM-1. Only 2 isolates contained OXA-58, one of which co-harboured NDM-1 (Table 1).

## Discussion

*A. baumannii* is a problematic pathogen in many hospitals and healthcare settings. Its epidemiology is particularly interesting in low- and middle income countries (LMICs) where outbreaks are commonly poly-clonal with diverse resistance mechanisms [16–18]. The most common global *A. baumannii* clone belongs to IC2/CC92 and contains OXA-23 as the most common acquired carbapenemase [19, 20]. This is typically seen in data reported from the Middle East and Egypt in particular [6, 9, 21]. The data presented at the NCI in Cairo shows a diverse epidemiology with several clones present simultaneously within the hospital, as seen by the number of different STs identified, which coincide with previous reports from Egypt showing that *A. baumannii* is predominantly poly-clonal and highly multi-drug resistant (MDR) [9]. ST208, part of IC2, is a recurring clone from 2013 to date, indicating the success and maintenance of IC2 in Egyptian hospitals.

One of the major concerns regarding *A. baumannii* infections is the rapid clonal spread in hospitals worldwide. Novel strains are continuously introduced to the clinical setting, which upon prolonged exposure to antibiotics, acquire and accumulate resistance genes rendering the infections untreatable. There is also an uncertainty of the natural habitat and potential reservoirs of *A. baumannii* is, but it seems that environmental and zoonotic prevalence should not be overlooked. Studies have identified *A. baumannii* in water, sewage and soil samples [22], as well as animal reservoirs such as cattle, pigs, sheep and birds [14, 23]. As reported in this study, 5 *A. baumannii* ST732 isolates harboured OXA-378 as the intrinsic OXA-51-like enzyme, which has been reported in stork samples from Poland and Germany [14]. The results furthermore show the continuous introduction of novel clones into the healthcare setting, and subsequently leads us to speculate on the role of migratory birds could have in the dispersion of *A. baumannii* globally. It is important to note however that environmental and zoonotic *A. baumannii* strains are usually sensitive to carbapenems, but the clones seem to acquire carbapenemases due to the antibiotic selection pressures in the clinical setting. In the current study the ST732/OXA-378 isolates contained OXA-23 and were carbapenem resistant. According to the pubMLST database, ST732 has only been reported in Portugal, and no other data is available. Further analysis revealed that ST732 is neither a SLV nor DVL of any other ST in the database, confirming that it is a rare clinical type.

Successful international clones, particularly IC2 are predominantly resistant due to OXA-23, as reflected in our data from the NCI. However, the poly-clonal dissemination of *A. baumannii* in the NCI suggests the introduction of multiple epidemiologically diverse isolates from unknown sources. OXA-510 is a new variant in the OXA-51-like family, and although the ST was unobtainable, it is a DLV of the IC2/CC92, which furthermore confirms the global success of IC2. The OXA-510 isolate is sensitive to carbapanem, but may acquire mobile resistance elements such as plasmids, which would contribute to its success and spread.

Carbapenem resistance was detected in 96.3% of the isolates. OXA-23 was the most common acquired carbapenemase, which is similar to previous global data. Metallo-β-lactamases VIM-1 and NDM-1 have also been identified co-harboured by several isolated in the study. Resistant isolates were not epidemiologically related, but is probably mediated by the presence of several resistance plasmids. Further work is underway to accurately characterise the presence of resistance plasmids in these isolates.

## Conclusion

We hereby present a poly-clonal dissemination of *A. baumannii* at the National Cancer Institute in Egypt. 93% of the isolates were carbapenem resistant and contained OXA-23, OXA-58, NDM-1 and VIM-1. 18 different STs were identified in the study, 5 of which are novel. The current study builds on previous work done in 2013 which looked at the diversity of *A. baumannii* in paediatric cancer patients in another hospital in Cairo [9, 24]. The results also showed poly-clonal dissemination, the predominance of IC2, and very high rates of resistance indicating the capability of *A. baumannii* persistence in the hospital environment through acquisition of several resistance genes. Together with similar results from Egypt, it is important that clinicians and healthcare workers are aware of the population of *A. baumannii* present in order to have appropriate treatment and infection control practices. It is also vital that molecular and genomic technologies are implemented in order to have an accurate epidemiological picture of *Acinetobacter sp.* in order to differentiate less pathogenic species and hospital-acquired persistors, particularly in centres serving high risk patients.

## Data Availability

Newly obtained sequences of *bla*_OXA-510_ was deposited in GenBank (www.ncbi.nlm.nih.gov/genbank) under accession number KU710721.1. All novel Sequence Types have been deposited in the PubMLST database (https://pubmlst.org/abaumannii/).

## Abbreviations

CC: Clonal Complex
CLSI: Clinical & Laboratory Standards Institute
DLV: Double Locus Variant
IC: International Clone
LMIC: Low- and Middle- Income Countries
MDR: Multidrug Resistant
MLST: Multilocus sequence typing
NCI: National Cancer Institute
SLV: Single Locus Variant
ST: Sequence Type
